# Stem Cells and Metastatic Cancer: Fatal Attraction?

**DOI:** 10.1371/journal.pmed.0030482

**Published:** 2006-12-26

**Authors:** Riccardo Fodde

## Abstract

Fodde discusses a new study in PLoS ONE, by Aboody and colleagues, who report on the successful eradication of whole-body disseminated metastases in a mouse model of neuroblastoma.

Neural stem cells (NSCs) are generally defined by their dual capacity to self-renew and differentiate into more specialized cell types such as neurons and glia. NSCs have been the object of many studies aimed at neuron replacement therapy in several degenerative conditions of the central nervous system such as Parkinson disease, Alzheimer disease, multiple sclerosis, and amyotrophic lateral sclerosis.

Pathotropism (movement towards diseased areas), a yet incompletely understood characteristic of NSCs, makes them particularly attractive candidates not only to replace damaged tissue in degenerative pathologies, but also to deliver therapeutic molecules in patients with disseminated metastatic cancer [1]. By 2000, several studies had shown that upon intracranial transplantation into animal models of brain cancer, NSCs are able to specifically migrate to sites of neoplasia [2–4], possibly in response to chemotactic signals emanating from cancer cells. Perhaps even more surprisingly, this tropism of neural stem cells can be exploited to target extracranial tumors of both neural and non-neural origins [5].

## Exploiting Pathotropism to Develop Treatments: A New *PLoS ONE* Study

In the launch issue of *PLoS ONE*, Aboody et al. report on the successful eradication of whole-body disseminated metastases in a mouse model of neuroblastoma [6]. The researchers took advantage of the tumor-tropic (selective migration towards cancer cells) properties of neural stem cells engineered to express an anti-cancer prodrug converting enzyme [6].

**Figure 1:**
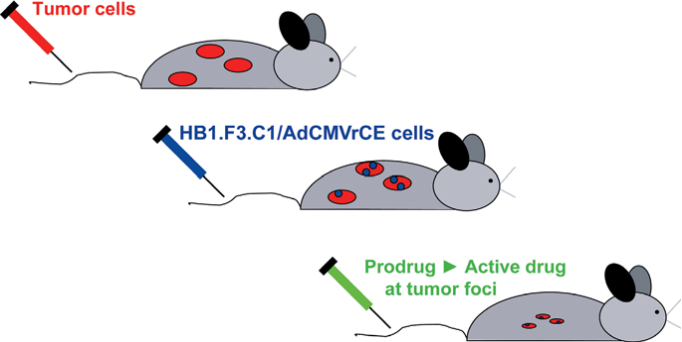
Schematic Diagram of the Protocol Used by Aboody and Colleagues From [6].

The experimental design they used is elegant in its straightforwardness (Figure 1). The tumor-tropic neural cell line HB1.F3.C1, originally derived by immortalization of human fetal telencephalon primary stem cells with a retrovirus encoding the v-*myc* oncogene [7], was employed as an in vivo delivery vector. To this aim, HB1.F3.C1 cells were engineered to encode for a secreted form of rabbit carboxylesterase (rCE), an enzyme capable of activating the anticancer prodrug CPT-11. Mice bearing disseminated neuroblastoma metastases were intravenously administrated with the modified NSCs and systemically treated with CPT-11. The in vivo–administered rCE-secreting stem cells selectively migrate to disseminated metastases where the enzyme activates the CPT-11 prodrug, thus increasing antitumor activity. Long-term (>6 months) monitoring of the treated animals showed 100% tumor-free survival when compared to control groups (50% survival).

These promising results point to the surprising ability of NSCs to selectively migrate to tumor sites independently of size and anatomical localization of the disseminated neuroblastoma metastases. Overall, the study by Aboody and collaborators lays a solid preclinical basis for experimental trials towards the eradication of disseminated metastatic disease in patients with cancer by intravenous administration of (neural) stem cells engineered to express therapeutic molecules. However, several research and safety issues should be considered.

## Research and Safety Issues

Ideally, (neural) stem cell lines used as in vivo vectors for the delivery of therapeutic molecules should retain the main features of both stem and progenitor cells, namely: (1) the capacity to differentiate in vivo in a predictable and controlled fashion, and (2) a sustained replication potential to allow propagation and expansion in vitro. The latter is usually achieved through immortalization by oncogene transformation, a procedure that raises obvious concerns about the potential tumorigenicity of the delivery vehicle.

From this point of view, the use of primary, rather than immortalized, stem cells may represent an attractive and safer alternative. However, in vitro expansion and genetic manipulation of primary stem cells is often cumbersome and not easily achieved, which represents a serious limitation for their use in the clinic. Alternatively, the identification of the growth factors underlying sustained replication potential in immortalized cells, and their implementation in the culture medium of primary (non-immortalized) stem cells, is likely to enhance their long-term and more efficient in vitro propagation. Notably, in contrast to mesenchymal stem cells, the v-*myc* immortalized HB1.F3.C1 NSCs do not replicate in vivo and are non-tumorigenic [7,8].

A second but equally relevant aspect is the stem cells' capacity to respond to disease-specific chemotactic signals. A limited number of stem cell attractants like VEGF (vascular endothelial growth factor), SDF1 (stromal cell-derived factor 1), and other cytokines emanating from brain pathologies have already been identified. However, it should be evident that additional and possibly disease-specific signals should be identified, together with the corresponding receptors on the cell surface of the stem cells used as therapeutic vehicles.

During the last few years, much effort has been devoted to the identification of cell surface markers for the prospective isolation of adult stem cells from several tissue-specific niches such as the mammary gland [9]. Analysis of the pathotropism of these primary cells or of cell lines derived from them is likely to provide additional and disease-specific stem cell vehicles to test in preclinical animal models.

Immunogenicity also represents a potential complication of any stem cell–based therapeutic application. While autologous stem cell sources represent the ideal solution, they can be a less practical option, especially if immortalization and/or further genetic manipulation of the vehicle cells are necessary. Although treatment with immune suppressive drugs may bypass the problem, additional studies are necessary to evaluate the true immunogenic potential of stem cells from allogeneic sources.

## Conclusion

Apart from the direct application for the treatment of metastatic diseases, the tropism of stem cells is likely to find applications in a broad spectrum of pathologies mainly depending on the migratory characteristics of the vehicle cells and of the chemoattractants emanating from the lesions in question. The main challenge ahead is to identify primary or immortalized stem cells from various niches for their capacity to migrate and localize at sites of focalized disease and deliver therapeutic molecules in situ without replicating in vivo.

## Abbreviations

NSC: neural stem cells
rCE: rabbit carboxylesterase
